# Identification of Serum Biomarkers Associated With Emergence Agitation After General Anesthesia in Adult Patients: A Metabolomics Analysis

**DOI:** 10.3389/fmed.2022.828867

**Published:** 2022-03-23

**Authors:** Xinning Mi, Jingshu Hong, Zhengqian Li, Taotao Liu, Qian Wang, Jiansuo Zhou, Yitong Li, Xiaoxiao Wang, Yi Yuan, Ning Yang, Yongzheng Han, Yang Zhou, Xiangyang Guo, Yue Li, Dengyang Han

**Affiliations:** ^1^Department of Anesthesiology, Peking University Third Hospital, Beijing, China; ^2^Department of Laboratory Medicine, Peking University Third Hospital, Beijing, China; ^3^Research Center of Clinical Epidemiology, Peking University Third Hospital, Beijing, China; ^4^Department of Anesthesiology, Beijing Jishuitan Hospital, Beijing, China

**Keywords:** emergence agitation, serum-matched metabolomics, general anesthesia, pathogenesis, decanoylcarnitine

## Abstract

**Background:**

Emergence agitation (EA) is a conscious disturbance after general anesthesia in adult patients that can lead to severe respiratory or circulatory complications and serious physical injury to patients and caregivers. However, the pathophysiological mechanisms underlying EA remain unclear. The present study aimed to identify serum metabolites with significant alterations in EA patients after general anesthesia and enable inferences on their associations with EA.

**Methods:**

EA patients were identified by Richmond Agitation-Sedation Scale (RASS) ≥ + 2 among a cohort of adult patients who received elective surgery under general anesthesia in Peking University Third Hospital between 01 June 2020 and 30 December 2020. We further selected sex-, age-, and surgery type-matched non-EA control patients at a 1:1.5 ratio. Postoperative serum samples were collected from both groups of patients. An untargeted metabolic method was used to identify differences in serum metabolomic profiles between the EA patients and the non-EA patients.

**Results:**

A total of 19 EA patients and 32 matched non-EA patients were included in the study. After screening and mapping with a database, 12 metabolites showed significant postoperative alterations in EA patients compared with non-EA patients, and were mainly involved in lipid, fatty acid and amino acid metabolism pathways. Receiver operating characteristic curve analyses indicated that vanillic acid, candoxatril, tiglylglycine, 5-methoxysalicylic acid, decanoylcarnitine, and 24-epibrassinolide may be involved in EA pathogenesis after general anesthesia.

**Conclusion:**

In this study, we found differences in the serum levels of vanillic acid, candoxatril, tiglylglycine, 5-methoxysalicylic acid, decanoylcarnitine, and 24-epibrassinolide involved in fatty acid metabolism, lipid metabolism, and amino acid metabolism pathways in EA patients compared with non-EA patients, which may demonstrate an EA pathogenesis-associated molecular pattern and contribute toward better understanding of EA occurrence.

## Introduction

Emergence agitation (EA) is an acute complication after general anesthesia characterized by restlessness, confusion, and possible combative or violent actions that occur as manifestations of conscious disturbance before patients totally come around and may have several adverse consequences (1). The incidence of EA can vary widely, depending on age, surgery type, anesthetic drugs used, assessment tools, and research methods (2–5). The reported incidence of EA is 4.7–22.2% in adults (6) and 10–80% in children (7). EA is a self-limited state with varying duration that shows spontaneous remission after patients become totally awake. The clinical manifestations of EA include physical and mental symptoms, such as rough actions and violent or agitated emotions. Although the symptoms are self-limited, the accompanying tachycardia, hypertension, and violent body movements, possibly with unintended extubation or accidental removal of catheters or drains during agitation, can cause severe respiratory or circulatory complications and serious physical injury (8). Therefore, it is necessary to reduce the incidence of EA and to recognize its occurrence in a timely manner to provide relevant protections if necessary. Existing studies on EA have mainly focused on pediatric patients, and there are few studies on adult patients. The limited available data suggest that male sex, smoking, urinary catheter, and postoperative pain are risk factors for EA, while age, inhalational anesthesia, history of substance misuse, and use of benzodiazepines during surgery are possible risk factors for EA (8). However, the pathogenesis of adult EA remains unclear, and clarification of its underlying mechanisms will be of benefit for the management of these patients.

Metabolomics is defined as a comprehensive analysis that can detect small molecular metabolites in biological samples under specific conditions with high sensitivity and good reproducibility. It is widely used to examine metabolites in studies that aim to identify biomarkers and provide possible molecular mechanisms for particular diseases, such as cancers, diabetes, neurodegenerative diseases, and perioperative neurocognitive disorders (9–13). Nevertheless, few studies have employed metabolomics analyses to examine EA in adult patients after general anesthesia. We had previously performed a metabolomic study to explore the correlation between the preoperative serum level of metabolites and postoperative EA (14). Several altered metabolites in serum before surgery may have predictive value for EA diagnosis. In this series study, we applied metabolomics methods based on liquid-phase chromatography-mass spectrometry (LC-MS) to determine the differences in postoperative serum metabolites between EA and non-EA patients, with the aim of identifying serum biomarkers associated with EA occurrence and clarifying the possible pathogenesis of EA after general anesthesia.

## Materials and Methods

### Ethics Approval and Clinical Registration

This retrospective nested case-control study was approved by the Peking University Third Hospital Medical Science Research Ethics Committee (Approval No. 2020-189-02) and registered at the Chinese Clinical Trial Registry (ChiCTR2000033911). All patients provided informed consent for the collection and analysis of their clinical data and serum samples.

### Study Population

**FIGURE 1:**
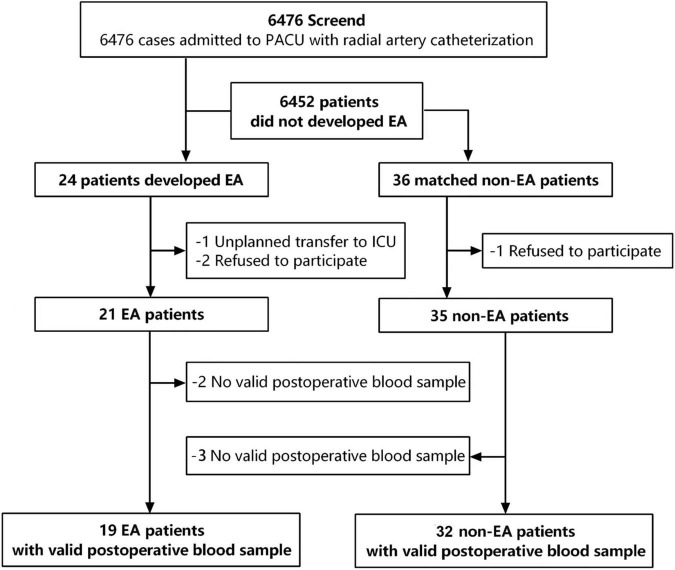
Flow chart of the research design. 6,476 patients were screened for emergency agitation, and 24 patients developed EA. 36 non-EA patients were matched with a ratio of 1:1.5 according to gender, age, BSA, and surgery types. Finally, 19 valid preoperative blood samples in the EA group and 32 valid preoperative blood samples in the non-EA group were collected for further analyses.

The study was conducted at Peking University Third Hospital between 01 June 2020 and 30 December 2020. Eligible patients were >18 years of age, had American Society of Anesthesiologists (ASA) physical class of I–III, received elective surgery under general anesthesia with radial artery catheterized pre-anesthesia for constant monitoring of arterial blood pressure, and were transferred to the postanesthesia care unit (PACU) after surgery, which shared the same population as previously described (14). A total of 6,476 patients were admitted to the PACU with radial artery catheterization during the study period, of whom 24 patients developed EA evaluated by Richmond Agitation-Sedation Scale (RASS) ≥ + 2. An individual-matching approach was then used to identify sex-, age-, and surgery type-matched control patients in a 1:1.5 ratio among the 6,452 patients who did not develop EA, and 36 patients were selected as the non-EA group. Patients were excluded if they: (1) had unplanned transfer to the ICU; (2) refused to participate; and (3) had invalid postoperative blood samples. Finally, 19 EA patients and 32 matched non-EA patients with valid postoperative blood samples were used for the analysis. The selection process is shown in Figure 1.

### Emergence Agitation Evaluation and Treatment

Patients were transferred to the PACU after surgery and assessed by a specified nurse anesthetist who was well-trained in use of the RAAS (Table 1) and identification of EA. Patients were diagnosed with EA if they had RASS ≥ + 2 (15, 16). All EA patients received appropriate treatment after being diagnosed. Specifically, if patients were recognized to have respiratory obstruction, hypoxia, or unstable hemodynamics, they were given relevant treatment as soon as possible. Activity was limited to prevent accidental injury or falling from the bed. Patients with slight agitation were called by their name to help them come around, asked if they felt uncomfortable, and given corresponding treatment or verbal consolation. Patients with severe agitation were administered low-dose propofol or sufentanil for rapid sedation and repeat treatment was permissible if necessary until their agitation ceased. All EA patients were returned to the ward after they became fully awake and were assessed to meet the discharge criteria.

**TABLE 1:** Richmond agitation and sedation scale (RAAS).

Score	Term	Description
+4	Combative	Overtly combative or violent; immediate danger to staff
+3	Very agitated	Pulls on or removes tube(s) or catheter(s) or has aggressive behavior
+2	Agitated	Frequent non-purposeful movement or patient–ventilator dyssynchrony
+1	Restless	Anxious or apprehensive but movements not aggressive or vigorous
0	Alert and calm	
–1	Drowsy	Not fully alert, but has sustained (> 10 s) awakening
–2	Light sedation	Briefly (<10 s) awakens with eye contact to voice
–3	Moderate sedation	Any movement (but no eye contact) to voice
–4	Deep sedation	No response to voice, but any movement to physical stimulation
–5	Unarousable	No response to voice or physical stimulation

### Clinical Characteristics Collection

The general clinical characteristics of the eligible patients (age, sex, height, weight, diagnosis, complications, surgery type, ASA physical class, preanesthetic medication, surgery duration, anesthesia duration, bleeding volume, blood transfusion, analgesic strategy, PACU residence duration) were collected from their medical records.

### Sample Collection

All patients underwent radial artery catheter placement under local anesthesia with 2% lidocaine before general anesthesia was conducted. For the EA patients, 2-mL arterial blood samples were collected during agitation. For the non-EA patients, 2-mL arterial blood samples were collected after they became totally awake and before they returned to the ward. All blood samples were centrifuged at 3,000 rpm (1,000 × *g*) for 10 min at 4°C to prepare serum samples according to the instructions. The serum samples were labeled and stored at –80°C until further analysis.

### Metabolites Extraction

Based on previous studies (17, 18), a 100-μL aliquot of each serum sample was added to 400 μL of extract solution (methanol

and acetonitrile mixture at a volume ratio of 1:1) containing an isotopically labeled internal standard substance. The mixtures were then vortexed for 30 s, sonicated for 10 min in an ice-water bath, and incubated for 1 h at –40°C to precipitate the proteins. After centrifugation at 12,000 rpm for 15 min at 4°C, equal aliquots of all supernatants were mixed to prepare a quality control sample for the analysis.

### Metabolic Profiling Analysis

LC-MS/MS analysis was performed using a UHPLC system (Vanquish; Thermo Fisher Scientific, Waltham, MA, United States) with a UPLC BEH Amide column (2.1 mm × 100 mm × 1.7 μm) coupled with a QExactive HFX mass spectrometer (Orbitrap MS; Thermo Scientific) (19). The mobile phase consisted of phase A (aqueous phase; mixture of 25 mmol/L ammonium acetate and 25 mmol/L ammonia hydroxide with pH 9.75) and phase B (acetonitrile). The elution gradient procedure was as follows: 0–0.5 min, 95% B; 0.5–7.0 min, 95–65% B; 7.0–8.0 min, 65–40% B; 8.0–9.0 min, 40% B; 9.0–9.1 min, 40–95% B; 9.1–12.0 min, 95% B. The flow velocity of the mobile phase was set at 0.5 mL/min. The column temperature was 30°C. The auto-sampler temperature was 4°C, and the injection volume was 3 μL. The QExactive HFX mass spectrometer was used to acquire MS/MS spectra in the information-dependent acquisition (IDA) mode and to continuously evaluate the full-scan MS spectrum under control of the acquisition software (Xcalibur; Thermo Fisher Scientific). The ESI source conditions were as follows: sheath gas flow rate, 50 Arb; Aux gas flow rate, 10 Arb; capillary temperature, 320°C; full MS resolution, 60,000; MS/MS resolution, 7,500; collision energy, 10/30/60 in NCE mode; spray voltage, 3.5 kV (positive) or -3.2 kV (negative). The raw data from the LC-MS/MS analysis were converted to the mzXML format using ProteoWizard software.^1^ Next, an R program (R Foundation for Statistical Computing, Vienna, Austria) based on XCMS was used for peak detection, extraction, alignment, and integration. Finally, an in-house MS2 database named BiotreeDB (v2.1) was applied for metabolite annotation, with the cutoff for annotation set at 0.3 (20).

### Statistical Analysis

The data were presented as mean ± standard deviation (SD), proportion, and frequency. The Kolmogorov–Smirnov method was used to test the normality of the variables. Continuous variables with a normal distribution and skewed distribution were presented as mean ± SD and interquartile range, respectively. Categorical variables were expressed as frequency and proportion. Normally distributed categorical variables were analyzed using a two-independent-sample *t*-test and skewedly distributed categorical variables were analyzed using the Mann–Whitney *U*-test. The chi-square test was used to analyze categorical variables. SPSS software (v27.0; IBM Corp., Armonk, NY, United States) was used for these data analyses.

SIMCA-P software (v15.0.2; Sartorius Stedim Data Analytics AB, Umea, Sweden) was used for analyses of the multivariate data for the serum metabolites. An orthogonal partial least-squares discriminant analysis (OPLS-DA) model was used to evaluate the differences between the two groups. A permutation test was used to verify the overfitting of the model and two parameters were determined to reveal the quality of the model. Specifically, R2 and Q2 indicated the interpretation rate and predictive ability of the model, respectively. Metabolites with variable importance in projection (VIP) > 1 and *p* < 0.05 (Student’s *t*-test) were considered to show significant changes. Commercial databases, including KEGG^2^ and MetaboAnalyst,^3^ were used for pathway enrichment analysis.

## Results

### Demographic and Clinical Characteristics of the Emergence Agitation and Non-emergence Agitation Patients

A total of 52 patients were included in the study. Nineteen serum samples were collected from patients who developed EA and 32 matched serum samples were collected from patients without EA. There were no significant differences in age, sex, body mass index, ASA physical class, anesthesia duration, surgery duration, and surgery type between the EA and non-EA groups (Table 2).

**TABLE 2:** Patients characteristics and surgery types of the EA and Non-EA groups.

Characteristic	EA group (*n* = 19)	Non-EA group (*n* = 32)	*P*-value
RASS scores	+ 3 (+2, +4)	0 (0, 0)	<0.001
Age (yr)	64.2 ± 16.8	60.3 ± 10.3	0.293
Male/Female	15/4	24/8	0.748
BMI (kg/m^2^)	24.1 ± 2.9	24.0 ± 4.5	0.899
Surgery type			0.179
General	5	11	
Neurosurgical	4	1	
Thoracic	3	8	
Gynecologic	0	4	
Orthopedic	4	5	
Urologic	3	3	

*RASS, Richmond Agitation Sedation Scale; BMI, Body Mass Index.*

### Distinct Clustering of Metabolites in the Emergence Agitation Group vs. the Non-emergence Agitation Group

**FIGURE 2:**
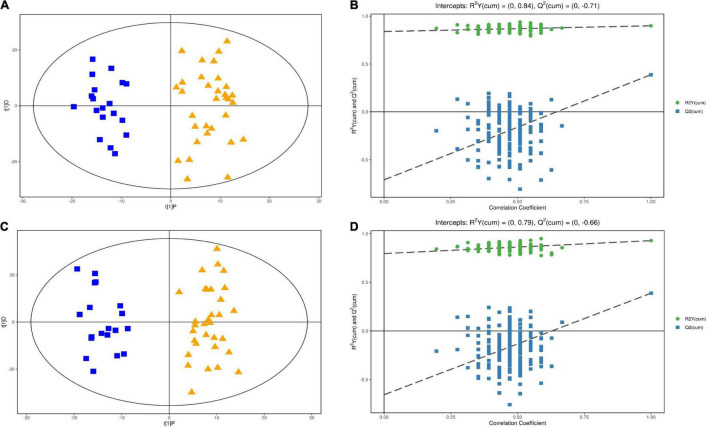
Multivariable analysis and selection of discriminant variables with both positive and negative ion modes between the EA group and the non-EA group. **(A,C)** Revealed the OPLS-DA score plots with further permutation tests **(B)** in the positive mode and **(D)** in the negative mode] between the two groups.

We utilized LC-MS/MS analysis to explore the serum metabolites that differed between the EA and non-EA patients. OPLS-DA score plots showed clear and distinctive clusters in the serum samples from the two groups in both the negative and positive ion modes (Figures 2A,C), indicating that metabolic differences existed between the groups. In the permutation analysis, permuted R2Y was 0.84 in the negative ion mode and 0.79 in the positive ion mode, while permuted Q2 was –0.71 in the negative ion mode and –0.66 in the positive ion mode (Figures 2B,D), indicating that the model fitting and prediction were valid.

### Differentially Expressed Metabolites Between the Emergence Agitation Group and the Non-emergence Agitation Group

**FIGURE 3:**
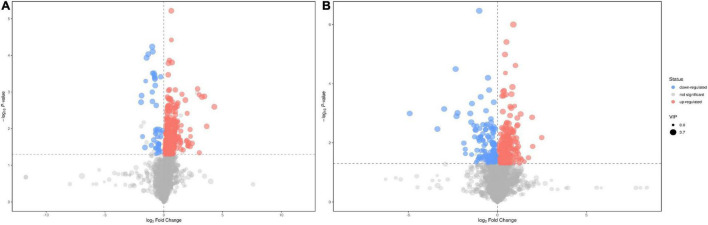
Volcano plots of differential features of metabolites between the EA group and the non-EA group. 428 features in the negative mode **(A)** and 363 features in the positive mode **(B)** were selected using the criteria of VIP > 1 and *p* < 0.05. As shown in the volcano plots, the increased and decreased features were marked as red and blue, respectively.

Based on the VIP values of the metabolites in the OPLS-DA model and the *t*-test for discrimination (VIP > 1 and *p* < 0.05), we screened 428 metabolites in the negative mode and 363 metabolites in the positive mode that were differentially expressed between the EA group and the non-EA group. The between-group differences in the metabolites in the negative and positive ion modes were plotted as volcano maps (Figures 3A,B), in which red dots represented up-regulated metabolites and blue dots represented down-regulated metabolites. The sizes of the dots represented their VIP values.

A total of 74 differentially expressed metabolites were identified in the serum samples from the EA and non-EA groups (Supplementary Table 1). Specifically, 61 metabolites (24 in the positive mode and 37 in the negative mode) were up-regulated and 13 metabolites (8 in the positive mode and 5 in the negative mode) were down-regulated in the EA group compared with the non-EA group.

**FIGURE 4:**
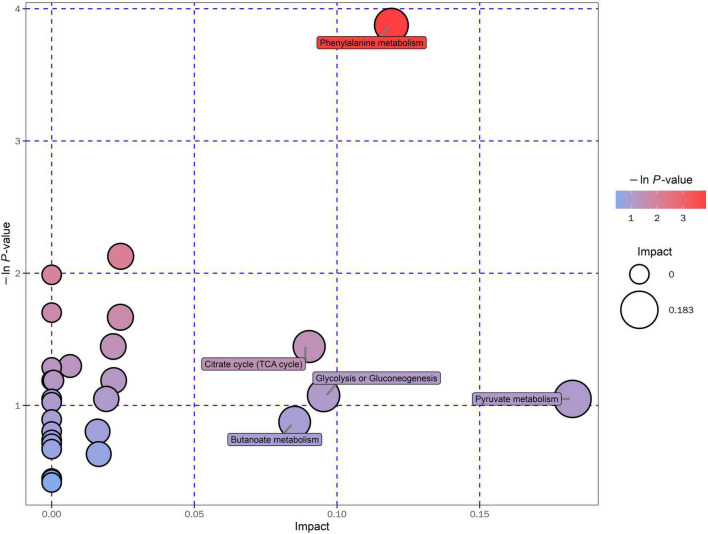
KEGG pathway enrichment analyses of the 12 altered metabolites between the EA group and the non-EA group. Each bubble represented a pathway, and 10 pathways were included in this bubble plot. The bubble size indicated the impact factor of the pathway in the topology analysis, and the bigger the size, the larger the impact factor. The bubble color represented the enrichment degree, and the deeper the color, the smaller the *P*-value, indicating more significant enrichment.

After peak alignment with the MS database and screening, a total of 12 metabolites were identified to be differentially expressed between the EA and non-EA groups (Table 3). The 12 metabolites belonged to the benzenoids (5), lipids and lipid-like molecules (4), organic acids and derivatives (2), and organohalogen compounds (1). The differentially expressed metabolites were mainly involved in fatty acid metabolism, lipid metabolism, and amino acid metabolism pathways according to the KEGG and MetaboAnalyst databases. The pathway enrichment of the 12 significantly altered metabolites was further analyzed and 10 KEGG pathways were identified, of which the metabolic pathways are shown in Figure 4.

**TABLE 3:** Differentially expressed metabolites in the serum of EA and non-EA patients under general anesthesia.

Metabolites	ESI^±^	Super class	VIP	*FC*	*P*-value	Trend
Dibutyl phthalate	–	Benzenoids	1.50	8.10	0.001	Up
5-Methoxysalicylic acid	–	Benzenoids	2.17	5.31	0.025	Up
Vanillic acid	–	Benzenoids	2.58	3.57	0.002	Up
Tiglylglycine	–	Organic acids and derivatives	2.67	2.31	0.017	Up
Perfluorooctanesulfonic acid	–	Organohalogen compounds	1.78	2.21	0.008	Up
24-Epibrassinolide	–	Lipids and lipid-like molecules	2.14	2.00	0.004	up
Gamma-Linolenic acid	–	Lipids and lipid-like molecules	1.74	0.48	0.028	Down
Phenylbutazone	–	Benzenoids	2.50	0.27	0.001	Down
L-alpha-Aspartyl-L-hydroxyproline	+	Organic acids and derivatives	2.16	2.16	0.020	Up
Decanoylcarnitine	+	Lipids and lipid-like molecules	2.57	2.14	0.015	Up
Taurochenodeoxycholate-7-sulfate	+	Lipids and lipid-like molecules	2.06	0.50	0.028	Up
Candoxatril	+	Benzenoids	2.78	0.13	0.001	Up

*ESI, electrospray ionization; VIP, variable importance in the projection; FC, fold change.*

### Evaluation of Potential Biomarkers Related to Emergence Agitation in Serum

**FIGURE 5:**
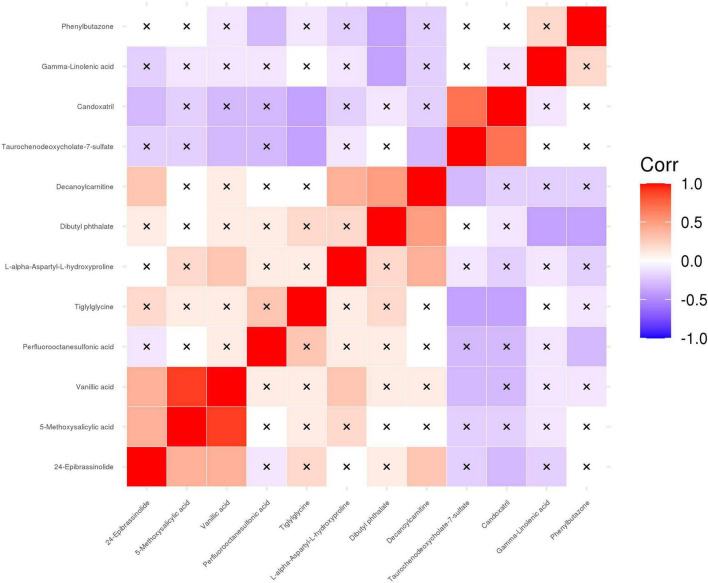
Correlation analyses of differential metabolites between the EA group and the non-EA group. 12 altered metabolites listed in Table 3 were performed Pearson correlation analyses using a heat map. The positive and negative correlations were shown in red and blue, respectively. A cross mark indicated a non-significant correlation.

**FIGURE 6:**
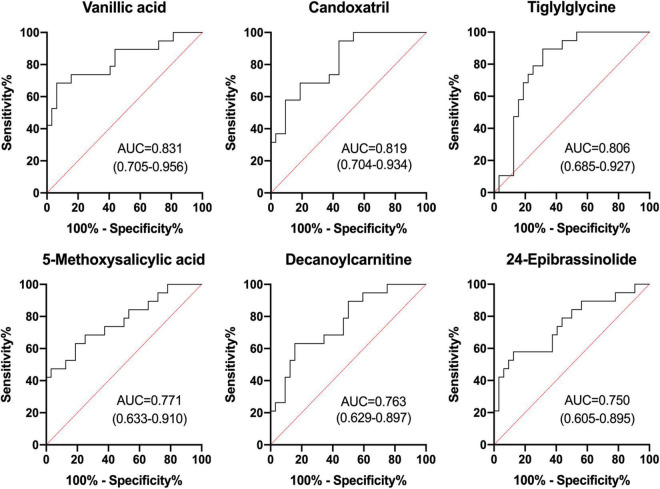
ROC analyses of selected differential metabolites in EA.

After correlation analyses (Figure 5), decanoylcarnitine, tiglylglycine, and vanillic acid were found to be closely related to other metabolites. The metabolites screened in Table 3 were examined by receiver operating characteristic (ROC) curve analyses and the six metabolites with the highest area under the curve (AUC) values were vanillic acid, candoxatril, tiglylglycine, 5-methoxysalicylic acid, decanoylcarnitine, and 24-epibrassinolide (Figure 6). Among them, vanillic acid, candoxatril, and tiglylglycine had AUC values of > 0.8, suggesting that these metabolites play important roles in the mechanism of EA after general anesthesia.

## Discussion

EA can lead to clinically significant consequences. However, its mechanisms remain unclear. It has been hypothesized that age, male sex, surgery type, long surgery duration, perioperative medications, postoperative pain, and presence of invasive monitors may contribute to EA (21). The underlying mechanisms for EA may be associated with various internal (e.g., nervous tension, anxiety) or external (e.g., surgical pain) stimuli that activate the underblunted sympathetic activity as consciousness gradually returns (1). However, a deeper understanding of the pivotal signaling molecules in EA remains in its infancy. The focus of the present study was to identify serum metabolite profiles associated with EA and to explore the mechanisms for EA pathogenesis and development.

LC-MS has obvious advantages for repeatability, sensitivity, and metabolite coverage, and has become the most commonly used method for high-throughput metabolic analyses (17, 22). Therefore, in the present study, we employed LC-MS to determine the different metabolite patterns in serum samples from EA patients and non-EA patients, with the aim of identifying differentially expressed serological metabolites associated with EA and further exploring EA pathogenesis. We identified a total of 12 metabolites that were differentially expressed between the EA and non-EA groups with AUC values of > 0.7 in the ROC analyses, and were mainly involved in fatty acid metabolism, lipid metabolism, and amino acid metabolism pathways. These altered metabolites suggest that perturbed metabolic processes may underlie the pathology of EA, but may also contribute to prevention of EA and reduction of perioperative complications.

Decanoylcarnitine belongs to the medium-chain acylcarnitines that play important roles in long-chain fatty acid metabolism by serving as carriers that transport activated long-chain fatty acids into mitochondria and transport coenzyme-A across the inner mitochondrial membrane for β-oxidation to provide energy for cellular functions (23–25). Besides fatty acid metabolism, acylcarnitines are also thought to be involved in other essential physiological processes such as cholinergic transmission because of their comparable chemical structures to acetylcholine (26). In the present study, we found that decanoylcarnitine was increased in the serum samples from EA patients, indicating that fatty acid metabolism pathways involved with energy bioenergetics and cholinergic pathways participate in the mechanism of EA. Previous metabolomics analyses on patients with Alzheimer’s disease and schizophrenia also found changes in acylcarnitines in their serum and brain samples (23, 27–29). Because acylcarnitines can be transported through the blood-brain barrier (26), it is supposed that changes in the serum decanoylcarnitine level could be responsible for the neurological symptoms of EA.

Tiglylglycine is a byproduct of the isoleucine degradation pathway. During the degradation of isoleucine, tiglylglycine is formed by the mitochondrial complex-1 enzyme. Another degradation product of isoleucine is acetyl-CoA, a branched-chain amino acid that ultimately feeds into the tricarboxylic acid cycle, suggesting the probable of TCA and glycolysis pathway involving in the mechanism of EA (30, 31). In the present study, we newly found that tiglylglycine was increased in the serum of EA patients during agitation, which has rarely been found in neurological diseases before. The observed increase is indicative of complex-1 dysfunction because isoleucine is not fully broken down to acetyl-CoA and instead converted to tiglylglycine (32). The increase in tiglylglycine revealed in this study highlights a potential mechanism for EA involving mitochondrial dysfunction and energy metabolism-related pathways.

Vanillic acid is a benzoic acid derivative, and can be converted from vanillylmandelic acid or homovanillic acid associated with the catecholamine pathway. Vanillic acid was reported to have antioxidant, anti-inflammatory, and neuroprotective effects in rodent brains (33, 34). Although the specific mechanism for how vanillic acid becomes increased in the serum of EA patients was not explored in the present study, a previous metabolomics analysis similarly found that the serum concentration of vanillic acid could be used to discriminate between patients with Parkinson’s disease and control patients (35). The possibility that occurrence of EA may be associated with preoperative existing Parkinson’s disease needs further research.

Candoxatril, a neutral endopeptidase inhibitor, is often used to treat cardiovascular diseases because it exerts a blood pressure-lowering effect. A study found that candoxatril was related to decreased plasma triglyceride and insulin levels, indicating that candoxatril was involved in some metabolic dysfunction (36). The present study found that candoxatril was increased in the serum of EA patients, suggesting that low blood pressure during surgery and triglyceride-related metabolic dysfunction may be relevant to EA.

The differentially expressed metabolites between EA and non-EA patients identified in the present study participate in fatty acid metabolism, lipid metabolism, and amino acid metabolism pathways, indicating that complicated molecular mechanisms underlie EA, including cholinergic system dysfunction, mitochondrial abnormality, and energy metabolic dysfunction, as well as probable oxidative stress and neuroinflammatory mechanisms. The identified metabolites were also found to be differentially expressed in neurodegenerative diseases such as Parkinson’s disease, dementia, cognitive decline, and mental disorders including schizophrenia and depression (37), as mentioned above, suggesting that similar metabolic patterns exist among these diseases and EA. The possibility that preoperative existing clinical or subclinical neurodegenerative diseases and mental disorders may be associated with EA occurrence remains to be investigated.

Although this study identified differentially expressed metabolites in EA patients and predicted their possible roles in EA, there are also some limitations. First, the sample size was not sufficiently large because of the low incidence rate of EA in adults. A larger sample size should be investigated to consolidate the conclusions reached in the present study. Second, additional verification and functional studies should be performed after the untargeted metabolomics analyses, and the potential mechanism of EA should be further elucidated.

## Conclusion

In conclusion, we performed a nested case-control study using a metabolomics method based on LC-MS/MS to reveal the metabolite differences in EA and established a different postoperative serum metabolite pattern in EA patients compared with non-EA patients. Specifically, we found different serum levels of vanillic acid, candoxatril, tiglylglycine, 5-methoxysalicylic acid, decanoylcarnitine, and 24-epibrassinolide involved in fatty acid metabolism, lipid metabolism, and amino acid metabolism pathways, suggesting that cholinergic system dysfunction, mitochondrial abnormality, and energy metabolic dysfunction, as well as probable oxidative stress and neuroinflammatory mechanisms, may play a part in EA pathogenesis. Analyses of ROC curves for several metabolites were employed to further evaluate their importance and reliability in EA pathogenesis. Verification of the molecular biology and metabolic function of the altered metabolites should be undertaken in future studies to deepen the understanding of how EA occurs.

## Data Availability Statement

The datasets presented in this study can be found in online repositories. The names of the repository/repositories and accession number(s) can be found below: Metabolights, MTBLS4227.

## Ethics Statement

The studies involving human participants were reviewed and approved by the Peking University Third Hospital Medical Science Research Ethics Committee (Approval No. 2020-189-02). The patients/participants provided their written informed consent to participate in this study.

## Author Contributions

DH and YuL: conceptualization and writing—review and editing. XM and JH: data curation. ZL, TL, QW, JZ, and YiL: investigation. XW and YY: methodology. YZ and XG: project administration. NY and YH: resources. DH: supervision. XM: writing—original draft. All authors contributed to the article and approved the submitted version.

## Conflict of Interest

The authors declare that the research was conducted in the absence of any commercial or financial relationships that could be construed as a potential conflict of interest.

## Publisher’s Note

All claims expressed in this article are solely those of the authors and do not necessarily represent those of their affiliated organizations, or those of the publisher, the editors and the reviewers. Any product that may be evaluated in this article, or claim that may be made by its manufacturer, is not guaranteed or endorsed by the publisher.

## Footnotes

1
http://proteowizard.sourceforge.net/

2
http://www.genome.jp/keg

3
http://www.metaboanalyst.ca/
