# Supplementary material for: Identification of Serum Biomarkers Associated With Emergence Agitation After General Anesthesia in Adult Patients: A Metabolomics Analysis
Source: Front Med (Lausanne). 2022 Mar 23;9:828867. doi: 10.3389/fmed.2022.828867 (PMC8983911; doi:10.3389/fmed.2022.828867)
Supplement: Supplementary file 1 [file Table_1.pdf]

Supplementary Table 1. 74 differentially expressed metabolites in the serum of EA and non-EA patients.

Metabolites	$ESI^{+/-}$	Super Class	VIP	FC	P-value
Threonic acid	negative	Organic oxygen compounds	2.88	1.61	0.00
Tiglylglycine	negative	Organic acids and derivatives	2.67	2.31	0.02
4-Dodecylbenzenesulfonic Acid	negative	Benzenoids	2.65	0.59	0.00
Vanillic acid	negative	Benzenoids	2.58	3.57	0.00
N-Acetyl-L-phenylalanine	negative	Organic acids and derivatives	2.57	1.56	0.00
Phenylbutazone	negative	Benzenoids	2.50	0.27	0.00
Creatinine	negative	Organic acids and derivatives	2.46	1.39	0.00
myo-Inositol	negative	Organic oxygen compounds	2.36	1.44	0.00
Palmitoleic acid	negative	Lipids and lipid-like molecules	2.34	1.84	0.02
Pseudouridine	negative	Nucleosides, nucleotides, and analo	2.21	1.25	0.00
5-Methoxysalicylic acid	negative	Benzenoids	2.17	5.31	0.03
Sunitinib	negative	Organoheterocyclic compounds	2.17	1.92	0.01
24-Epibrassinolide	negative	Lipids and lipid-like molecules	2.14	2.00	0.00
2-Hydroxy-3-methylbutyric acid	negative	Lipids and lipid-like molecules	2.10	1.32	0.01
Pyruvic acid	negative	Organic acids and derivatives	2.02	1.24	0.01
L-Phenylalanine	negative	Organic acids and derivatives	1.90	1.24	0.01
Ethyl stearate	negative	Lipids and lipid-like molecules	1.88	0.75	0.03
5Z-Dodecenoic acid	negative	Lipids and lipid-like molecules	1.81	1.55	0.05
3-Hydroxyisovaleric acid	negative	Lipids and lipid-like molecules	1.80	1.30	0.04
Deoxycholic acid	negative	Lipids and lipid-like molecules	1.80	1.50	0.03
Perfluorooctanesulfonic acid	negative	Organohalogen compounds	1.78	2.21	0.01

Ribothymidine	negative	Nucleosides, nucleotides, and analo	1.78	1.18	0.01
Nitrofurazone	negative	Organoheterocyclic compounds	1.78	1.20	0.04
Ethyl dodecanoate	negative	Lipids and lipid-like molecules	1.77	1.38	0.03
Gamma-Linolenic acid	negative	Lipids and lipid-like molecules	1.74	0.48	0.03
N-Acetyl-L-methionine	negative	Organic acids and derivatives	1.72	1.54	0.00
Esculentic acid (Diplazium)	negative	Lipids and lipid-like molecules	1.69	1.58	0.02
Glyceraldehyde	negative	Organic oxygen compounds	1.66	1.26	0.05
Terephthalic acid	negative	Benzenoids	1.63	1.07	0.05
L-3-Phenyllactic acid	negative	Phenylpropanoids and polyketides	1.63	1.96	0.02
9-Decenoic acid	negative	Lipids and lipid-like molecules	1.60	1.29	0.02
Myristic acid	negative	Lipids and lipid-like molecules	1.58	1.20	0.01
Deoxyuridine	negative	Nucleosides, nucleotides, and analo	1.58	1.31	0.02
Ethyl oleate	negative	Lipids and lipid-like molecules	1.55	1.45	0.01
Dibutyl phthalate	negative	Benzenoids	1.50	8.10	0.00
L-Norleucine	negative	Organic acids and derivatives	1.50	1.18	0.04
Cerulenin	negative	Organoheterocyclic compounds	1.35	1.89	0.03
2-Ketohexanoic acid	negative	Organic acids and derivatives	1.35	1.28	0.04
LysoPE(18:1(9Z)/0:0)	negative	Lipids and lipid-like molecules	1.32	0.73	0.03
3-Methoxy-4-hydroxyphenylethylen	negative	Organic acids and derivatives	1.26	1.74	0.05
N-Acetyl-L-alanine	negative	Organic acids and derivatives	1.16	1.30	0.00
Succinic anhydride	negative	Organoheterocyclic compounds	1.14	1.78	0.04
Creatinine	positive	Organic acids and derivatives	2.05	1.16	0.02
L-Gulose	positive	Organic oxygen compounds	1.98	1.27	0.01

D-Alanine	positive	Organic acids and derivatives	1.90	1.20	0.01
Isoleucyl-Alanine	positive	Organic acids and derivatives	1.38	0.51	0.00
Kynurenic acid	positive	Organoheterocyclic compounds	2.55	1.64	0.00
N6-Methyladenosine	positive	Nucleosides, nucleotides, and analo	2.78	1.21	0.00
1-Methylhypoxanthine	positive	Organoheterocyclic compounds	2.13	1.32	0.02
3-Methylguanine	positive	Organoheterocyclic compounds	2.23	1.20	0.02
N-Acetylhistidine	positive	Organic acids and derivatives	2.00	1.54	0.01
5-Ethyl-2,4-dimethyloxazole	positive	Organoheterocyclic compounds	1.67	1.39	0.01
Pirbuterol	positive	Organoheterocyclic compounds	2.20	1.54	0.05
LysoPE(18:1(9Z)/0:0)	positive	Lipids and lipid-like molecules	1.69	0.69	0.04
Octadecylamine	positive	Organonitrogen compounds	1.85	0.70	0.01
Palmitoylethanolamide	positive	Organic acids and derivatives	1.28	1.40	0.04
5-Hydroxy-L-tryptophan	positive	Organoheterocyclic compounds	1.32	1.20	0.04
L-alpha-Aspartyl-L-hydroxyproline	positive	Organic acids and derivatives	2.16	2.16	0.02
2-Methylbutyroylcarnitine	positive	Lipids and lipid-like molecules	2.03	1.42	0.04
3-Dehydroxycarnitine	positive	Lipids and lipid-like molecules	2.32	1.44	0.01
Taurochenodeoxycholate-7-sulfate	positive	Lipids and lipid-like molecules	2.06	0.50	0.03
Formiminoglutamic acid	positive	Organic acids and derivatives	2.55	1.81	0.00
Epidermin	positive	Organic oxygen compounds	2.74	1.41	0.00
Candoxatril	positive	Benzenoids	2.78	0.13	0.00
Methylguanidine	positive	Organic nitrogen compounds	1.94	1.34	0.03
8-Deoxy-11-hydroxy-13-chlorogros	positive	Lipids and lipid-like molecules	1.02	1.89	0.03
Linamarin	positive	Organic oxygen compounds	2.39	1.38	0.03

PC(18:3(6Z,9Z,12Z)/20:4(5Z,8Z,11	positive	Lipids and lipid-like molecules	2.07	0.88	0.02
PE(P-18:1(11Z)/16:0)	positive	Lipids and lipid-like molecules	1.03	0.70	0.05
PC(20:2(11Z,14Z)/P-18:0)	positive	Lipids and lipid-like molecules	1.14	0.81	0.04
Decanoylcarnitine	positive	Lipids and lipid-like molecules	2.57	2.14	0.02
N2,N2-Dimethylguanosine	positive	Nucleosides, nucleotides, and analo	1.88	1.42	0.01
Sertraline	positive	Benzenoids	1.61	1.88	0.05
PC(22:6(4Z,7Z,10Z,13Z,16Z,19Z)/1	positive	Lipids and lipid-like molecules	1.64	1.13	0.01

ESI, electrospray ionization; VIP, variable importance in the projection; FC, fold change.
